# Perception and willingness toward second-generation long-acting antipsychotics among patients with schizophrenia: a cross-sectional survey in Fujian, China

**DOI:** 10.3389/fpsyt.2026.1841818

**Published:** 2026-07-14

**Authors:** Yixiang Zhou, Duoduo Lin, Haibin Zhang, Yinghua Huang

**Affiliations:** 1 Department of Pharmacy, Xiamen Xianyue Hospital, Xiamen, China; 2 Xianyue Hospital Affiliated with Xiamen Medical College, Xiamen, China; 3 Fujian Psychiatric Center, Xiamen, China; 4 Fujian Clinical Research Center for Mental Disorders, Xiamen, China; 5 Department of Psychiatry, Xiamen Xianyue Hospital, Xiamen, China

**Keywords:** cross-sectional survey, medication adherence, perception, schizophrenia, second-generation antipsychotic long-acting injection, willingness

## Abstract

**Objective:**

To assess perceptions and willingness toward second-generation antipsychotic long-acting injectables (SGA-LAIs) among patients with schizophrenia, to identify the influencing factors, and to provide evidence for optimizing clinical implementation strategies.

**Methods:**

A cross-sectional survey was conducted in which patients with schizophrenia who attended outpatient follow-up visits at a tertiary psychiatric hospital were recruited via convenience sampling. A validated, expert-reviewed questionnaire was administered. Data were analyzed using descriptive statistics and chi-square tests, with binary logistic regression models employed to explore the determinants of patients’ willingness to accept SGA-LAIs.

**Results:**

The sample characteristics included 126 males (55.5%), 114 patients (50.2%) aged 31–50 years, 140 patients (61.7%) with an illness course exceeding 5 years, and 126 patients (55.5%) with experience with SGA-LAIs. Overall, 155 participants (68.3%) expressed positive willingness to use SGA-LAIs, whereas 72 participants (31.7%) expressed negative willingness. Univariate analysis revealed significant differences between the willingness-positive and willingness-negative groups in terms of disease course (χ² = 12.314, *P* = 0.006) and prior SGA-LAI experience (χ² = 55.525, *P* < 0.001). Binary logistic regression analysis revealed that patients with prior SGA-LAI experience (OR = 11.11, 95% CI: 5.41~23.26, *P* < 0.001), a disease course of 5–10 years (OR = 2.898, 95% CI: 1.134-7.407, *P* = 0.026), and a disease course of > 10 years (OR = 3.282, 95% CI: 1.300-8.288, *P* = 0.012) demonstrated significantly greater willingness to accept SGA-LAIs. Among the willingness-positive group, the most endorsed advantages of SGA-LAIs were “avoiding daily medication” (80.7%) and “increased effectiveness” (68.4%), and the primary concern was “injection pain” (56.8%). In the willingness-negative group, the predominant reasons for refusal were “rejection of needles” (93.1%) and “rejection of changing therapeutic regimens” (79.2%).

**Conclusion:**

The overall acceptance of and willingness to use SGA-LAIs among patients with schizophrenia followed up in psychiatric hospitals are high. Experience with SGA-LAIs and disease course were identified as independent factors influencing patients’ willingness to use SGA-LAIs. In clinical practice, patient acceptance can be enhanced by strengthening doctor-patient communication, prioritizing medication education for patients with a long disease course, and improving patients’ initial experience with SGA-LAIs, thereby supporting broader clinical adoption of SGA-LAIs.

## 1 Introduction

Schizophrenia is a chronic and recurring severe mental disorder that affects approximately 1% of the global population over a lifetime (1). It is characterized by significant cognitive deficits and behavioral disturbances and imposes substantial clinical and economic burden on patients, families, and society (2). Epidemiological data from China indicate that the lifetime prevalence of schizophrenia is 0.6% (3), with 6.43 million individuals registered nationwide as having a severe mental illness, of which approximately 4.5 million are diagnosed with schizophrenia (4).

Antipsychotic medications constitute the cornerstone of both acute and maintenance treatment for schizophrenia. Consistent long-term pharmacotherapy is essential for effective relapse prevention (5). However, poor adherence to oral antipsychotics is widespread. Studies have reported that 54.5% to 80% of individuals with schizophrenia do not take their medication as prescribed (6, 7). Factors contributing to nonadherence include medication-related issues, disease characteristics, low income, reduced quality of life, and individual patient attributes (8). Nonadherence directly increases the risk of relapse, rehospitalization, and functional deterioration, thereby substantially increasing overall treatment expenses and societal costs (9–11). Adults with schizophrenia demonstrate higher healthcare resource utilization, incurring an additional $14,087 in healthcare costs, largely driven by $4,677 higher long-term care expenditures (12). Consequently, enhancing medication adherence is a critical strategy for preventing relapse and improving long-term outcomes in patients with schizophrenia (13). Long-acting injectable antipsychotics (LAIs), particularly second-generation antipsychotic long-acting injectables (SGA-LAIs), increase treatment continuity and stability through their unique pharmacokinetic properties, which reduce the dosing frequency and maintain stable plasma drug concentrations. As a core strategy to improve medication adherence and lower relapse risk, LAIs should be especially beneficial for patients with poor compliance (14).

Evidence has indicated that LAIs significantly decrease relapse rates, hospital readmissions, healthcare resource utilization, and caregiver burden among individuals with schizophrenia (15, 16). Moreover, formulations with extended dosing intervals help improve quality of life (17). For instance, paliperidone palmitate three-monthly offers advantages over paliperidone palmitate one-monthly by further alleviating stigma and family care pressure, decreasing the frequency and time costs associated with medical visits, and enhancing patient quality of life (18). Patients also express a preference for longer-interval injections over oral medications and monthly injections (19). Given the clinical benefits, treatment guidelines recommend LAIs for the comprehensive management of schizophrenia, particularly for patients with adherence challenges and a high risk of relapse (20). In China, LAIs are included in the National Essential Drug List and the Essential Medical Insurance Drug List (21, 22), and relevant policies explicitly recommend their use for patients with severe mental disorders who have limited family supervision and are at risk of incidents (23). The implementation of a government-sponsored free-LAI policy has been shown to increase LAI prescription rates to 18.6% and reduce rehospitalization rates by 52% (24).

Despite the recognized clinical and public health advantages of LAIs (25), their utilization in clinical practice within China remains suboptimal. Data indicate that LAI prescription rates are approximately 20% in Europe and the United States, with an average of 17.9% across Asian countries but only 0.66% in mainland China (26–28). Previous research has suggested that the limited clinical application of LAIs is not only attributable to formulation characteristics and health policies but also closely linked to the prescribing behaviors of healthcare providers, as well as the awareness and acceptance among patients and their families (29–32). While previous studies have primarily focused on community-managed patients with schizophrenia (33, 34), psychiatric outpatients may differ substantially in terms of illness insight, treatment experience, healthcare access, and exposure to clinician-led medication counseling. These differences may influence perceptions and acceptance of SGA-LAIs. However, evidence regarding awareness, willingness, and determinants of SGA-LAsI acceptance among psychiatric outpatients remains limited (35). Therefore, this study aimed to investigate these factors among outpatients with schizophrenia attending a tertiary psychiatric hospital. The findings provide an empirical basis for optimizing clinician-patient communication strategies and enhancing healthcare service policies related to SGA-LAIs, ultimately improving the long-term treatment outcomes for patients with schizophrenia.

## 2 Materials and methods

### 2.1 Study design and ethical approval

This investigation employed a cross-sectional questionnaire-based design. The study protocol received formal approval from the Medical Ethics Committee of Xiamen Xianyue Hospital (Ethics Approval Number: 2025-KY-058). All procedures were conducted in strict accordance with the principles outlined in the Declaration of Helsinki. Participants provided voluntary, written informed consent following a comprehensive explanation of the study. Before obtaining written informed consent, each potential participant’s capacity to provide consent was clinically assessed by the attending psychiatrist based on their ability to understand the study information, weigh the risks and benefits, and communicate a voluntary decision. Throughout the research process, participant privacy was rigorously safeguarded, and no personally identifiable information was collected. Furthermore, participants did not receive any financial compensation for their involvement.

### 2.2 Study subjects

The study subjects included individuals who were diagnosed with schizophrenia and who attended follow-up appointments at the psychiatric clinic of Xiamen Xianyue Hospital between July and September 2025. The inclusion criteria were as follows: 1) a confirmed diagnosis of schizophrenia based on the International Classification of Diseases, 10th Revision (ICD-10) criteria; 2) age 18 years or older; and 3) clinical stability as assessed by the attending physician, with the capacity to comprehend the survey’s content and procedures and the ability to cooperate in completing the questionnaire. Patients with no medication adjustment and no changes in psychopathology over the past 3 months were defined as clinically stable (36). The exclusion criterion was defined as an inability to cooperate and complete the survey because of factors such as age or significant cognitive or communication impairments.

### 2.3 Survey instruments and questionnaire design

For this study, a self-administered questionnaire was developed. The initial draft was developed on the basis of the Chinese healthcare system, integrating clinical diagnosis and treatment scenarios, medical insurance policies, and patient characteristics. It leveraged on established instruments such as the Medication Adherence Report Scale (MARS), the Drug Attitude Inventory (DAI), and the Multidimensional Scale of Perceived Social Support (MSPSS), as well as those used in previous studies conducted in China. To ensure content validity and clinical applicability, the questionnaire underwent three rounds of expert review and revision, followed by pilot testing, which resulted in the final version. This committee comprised three chief physicians in psychiatry, two psychopharmacologists, and one statistician. In the pilot test, we invited 10 clinically stable outpatients (5 male, 5 female; mean age 43.5 years; mean illness duration 7.4 years) to complete the questionnaire, focusing specifically on item clarity, relevance, and comprehensibility. The reliability analysis results of the questionnaire were expressed using Cronbach’s α coefficient. The Cronbach’s α coefficient for the attitude toward the SGA-LAIs part of the questionnaire was 0.860, indicating good reliability. Their iterative feedback culminated in the final formal questionnaire.

The formal questionnaire comprises four sections: Demographic characteristics:gender, age, marital status, education level, occupation, family economic status, source of medical costs, and guardian status. Social support: guardians’ relationship with patients, and the guardians’ care ability. Disease and treatment experience: disease course, history of hospitalization, outpatient follow-up adherence, oral medication adherence, satisfaction with the current treatment regimen, and prior experience with SGA-LAIs. Attitudes and willingness toward SGA-LAIs: perceptions of drug advantages, medication concerns, willingness to use SGA-LAIs, and reasons for refusal.

### 2.4 Investigation implementation and quality control

All the investigators were psychiatric medical staff who received standardized training. The training covered informed consent procedures, interpretation of questionnaire items, the survey process, and communication techniques to ensure consistent implementation across all the investigators. The survey was conducted during patient waiting times in the outpatient clinic. The respondents completed the questionnaire online via a QR code linked to the Wenjuanxing platform on WeChat, with an average completion time of approximately 12 to 14 minutes. For patients who experienced difficulty operating smartphones, the investigators assisted in completing the survey according to the respondents’ verbal responses while ensuring autonomous decision-making. During questionnaire administration, caregivers (e.g., family members or guardians) were allowed to be present at the participants’ request, but they were instructed not to influence the participant’s responses. In cases where participants had difficulty reading or understanding the questions, trained researchers provided standardized explanations without leading the response. To enhance data quality, logic verification rules and mandatory item prompts were embedded within the electronic questionnaire to minimize missing values and logical errors. Following data exportation, two researchers independently performed data cleaning and verification. This process involved eliminating invalid questionnaires characterized by patterned responses or missing key information, thereby ensuring the reliability and integrity of the final data set.

### 2.5 Statistical methods

Data analysis was performed using SPSS 26.0. Continuous variables that followed a normal distribution are presented as the mean ± standard deviation and were compared between groups using the independent samples t test. Categorical data are expressed as counts (percentages) and were compared using the Chi-square (χ²) test. For the analysis of willingness to use SGA-LAIs, patients were categorized into two groups: a positive willingness group (“very willing” or “willing”) and a negative willingness group (“uncertain” or “unwilling”). The no-experience group included individuals who reported having “never heard of SGA-LAIs” or having “heard of SGA-LAIs but never used them”, whereas the experienced group encompasses those who have “used in past treatments” or are “being treated with SGA-LAIs”. To identify independent factors associated with willingness to use SGA-LAIs, a binary logistic regression analysis was conducted. First, univariate analyses were performed to screen for variables showing a statistical trend (*P* < 0.10) between the willingness groups. These variables, along with clinically relevant factors forced into the model (age, history of hospitalization, and outpatient follow-up compliance), were entered as independent variables. The dependent variable was binary: willingness to use SGA-LAIs or not. The enter method was used to construct the logistic regression model. The results are were reported as odds ratios (ORs) with corresponding 95% confidence intervals (CIs). A two-sided significance level of α = 0.05 was applied for all the statistical tests.

## 3 Results

### 3.1 Demographic and clinical characteristics of the study subjects

The demographic characteristics of the patients diagnosed with schizophrenia are detailed in **Table 1**. Among the 227 participants, 126 (55.5%) were male. The majority of the participants (114, 50.2%) were aged between 31 and 50 years. In terms of marital status, the greatest proportions were married (46.7%) and unmarried (43.6%). A significant majority of the respondents (63.0%) were unemployed. Medical expenses were primarily covered by health insurance or government subsidies (56.4%). Guardians were most frequently parents or children (58.1%), and 90.7% of the participants reported good guardianship ability. With respect to clinical characteristics, 140 participants (61.7%) had an illness course exceeding five years. A history of hospitalization related to schizophrenia was reported by 63.0% of the participants. Regular outpatient follow-up was maintained by 63.9% of the participants, good oral medication compliance was observed in 63.4% of the participants, and satisfaction with the current treatment regimen was expressed by 64.3% of the participants. Additionally, 55.5% of the participants had prior experience with SGA-LAIs.

**Table 1:** Demographic characteristics, social support, treatment experience, and attitude toward SGA-LAIs among participants (n=227).

Characteristic	Frequency, *n(*%)			t/χ²	*P*
	Overall (*n* = 227)	Unwillingness *n*(%) (*n* = 72)	Willingness *n*(%) (*n* = 155)		
Gender *n*(%)				2.930	0.087
Male	126(55.5)	34(47.2)	92(59.4)		
Female	101(44.5)	38(52.8)	63(40.6)		
Age *n*(%)				1.236	0.744
18-30	48(21.1)	15(20.8)	33(21.3)		
31-50	114(50.2)	36(50)	78(50.3)		
51-65	54(23.8)	19(26.4)	35(22.6)		
>65	11(4.8)	2(2.8)	9(5.8)		
Marital status, n (%)				3.123	0.373
Unmarried	99(43.6)	35(48.6)	64(41.3)		
Married	106(46.7)	30(41.7)	76(49)		
Divorce	19(8.4)	5(6.9)	14(9)		
Widowed	3(1.3)	2(2.8)	1(0.6)		
Education level, n (%)				3.094	0.377
Primary school and below	64(28.2)	18(25)	46(29.7)		
Junior high school	65(28.6)	23(31.9)	42(27.1)		
Senior high school	49(21.6)	19(26.4)	30(19.4)		
Junior college or above	49(21.6)	12(16.7)	37(23.9)		
Occupation, n (%)				2.796	0.424
Student	9(4.0)	1(1.4)	8(5.2)		
Employed	66(29.1)	23(31.9)	43(27.7)		
Retired	9(4.0)	4(5.6)	5(3.2)		
Unemployed	143(63.0)	44(61.1)	99(63.9)		
Family financial resources, n (%)				5.995	0.112
Poor	52(22.9)	23(31.9)	29(18.7)		
Neutral	159(70)	43(59.7)	116(74.8)		
Good	12(5.3)	5(6.9)	7(4.5)		
Better	4(1.8)	1(1.4)	3(1.9)		
Source of medical expenses, n (%)				0.340	0.844
Own expense	87(38.3)	27(37.5)	60(38.7)		
Medical insurance	128(56.4)	42(58.3)	86(55.5)		
Others	12(5.3)	3(4.2)	9(5.8)		
Guardian relationship, *n* (%)				0.240	0.887
Parents or children	132(58.1)	43(59.7)	89(57.4)		
Spouse	53(23.3)	17(23.6)	36(23.2)		
Others	42(18.5)	12(16.7)	30(19.4)		
Care ability, *n* (%)				2.893	0.235
Good	62(27.3)	20(27.8)	42(27.1)		
Neutral	144(63.4)	42(58.3)	102(65.8)		
Poor	21(9.3)	10(13.9)	11(7.1)		
Disease course *n*(%)				12.314	0.006
<2 years	22(9.7)	8(11.1)	14(9)		
2–5 years	65(28.6)	18(25)	47(30.3)		
6–10 years	68(30)	13(18.1)	55(35.5)		
>10 years	72(31.7)	33(45.8)	39(25.2)		
Hospitalization experience *n*(%)				3.353	0.340
Never hospitalized	84(37)	23(31.9)	61(39.4)		
1–3 times	101(44.5)	31(43.1)	70(45.2)		
4–5 times	29(12.8)	13(18.1)	16(10.3)		
>6 times	13(5.7)	5(6.9)	8(5.2)		
Outpatient adherence, n (%)				1.404	0.236
Regular	145(63.9)	42(58.3)	103(66.5)		
Irregular	82(36.1)	30(41.7)	52(33.5)		
Antipsychotics adherence, n (%)				0.970	0.325
Low adherence	83(36.6)	23(31.9)	60(38.7)		
High adherence	144(63.4)	49(68.1)	95(61.3)		
Satisfied with current treatment *n*(%)				2.015	0.365
satisfied	146(64.3)	51(70.8)	95(61.3)		
Neutrally	72(31.7)	19(26.4)	53(34.2)		
dissatisfied	9(4)	2(2.8)	7(4.5)		
Experience with SGA-LAl *n*(%)				55.525	<0.001
No Experienced	101(44.5)	58(80.6)	43(27.7)		
Experienced	126(55.5)	14(19.4)	112(72.3)		

### 3.2 Univariate analysis

The univariate analysis revealed no statistically significant differences between the groups in terms of gender, age, marital status, education level, occupation, economic status, source of medical costs, guardian status, history of hospitalization, outpatient follow-up compliance, oral medication compliance, or satisfaction with current treatment(all *P*> 0.05), as presented in **Table 1**. However, significant differences were identified for two variables: disease course (χ^2^ = 12.314, *P* = 0.006)and prior experience with SGA-LAIs(χ^2^ = 55.525, *P* < 0.001).

### 3.3 Multivariate binary logistic regression analysis

A multivariate binary logistic regression analysis was conducted to identify independent predictors of patients’ willingness to use LAIs. The independent variables included those with *P* < 0.10 from the univariate analysis (sex, disease course, and and experience with SGA-LAIs use) and clinically mandated variables (age, history of hospitalization, and outpatient adherence). Willingness to use LAIs served as the dependent variable.The results of the omnibus test of the model coefficients were significant (χ² = 72.44, *P* < 0.001), indicating adequate model fit. The Hosmer-Lemeshow test indicated adequate model fit (χ² = 15.68, *P* = 0.047). The model explained 52.0% of the variance (Nagelkerke R² = 0.520) and correctly classified 81.1% of the participants (sensitivity for the unwilling group = 72.2%, specificity for the willing group = 85.2%). Collinearity diagnostics showed that all variance inflation factors (VIFs) were below 2.5, indicating no serious multicollinearity among the independent variables. The regression analysis results revealed that prior experience with SGA-LAIs and disease course were independent factors significantly associated with patients’ willingness to use SGA-LAIs (**Table 2**). Patients with prior SGA-LAIs experience demonstrated a significantly greater willingness to use (OR = 11.11, 95%CI: 5.41~23.26, *P* < 0.001) than those without experience did. With respect to disease course, using a disease course of less than 2 years as the reference, patients with a disease course of 5–10 years (OR = 2.90, 95% CI: 1.13–7.41, *P* = 0.026) and >10 years (OR = 3.28, 95% CI: 1.30–8.29, *P* = 0.012) had a significantly greater willingness to use SGA-LAIs. In contrast, those with a disease course of 2–5 years (OR = 1.78, 95% CI: 0.50–6.30, *P* = 0.39) did not differ significantly from that of the reference group. Sex (OR = 1.53, 95% CI: 0.762–3.08, *P* = 0.23), age group (overall P = 0.78), history of hospitalization (overall *P* = 0.71), and outpatient adherence (OR = 1.13, 95% CI: 0.57–2.26, *P* = 0.73) were not significantly or independently associated with willingness to use these agents.

**Table 2:** Binary logistic regression analysis of different influencing factors on attitude toward SGA-LAIs.

Characteristic	β	Std. error	Wald	*P*	OR (95% CI)
Gender					
Male*					
Female	0.427	0.356	1.433	0.231	1.53 (0.76–3.08)
Course of disease					
<2 years*					
2–5 years	0.571	0.648	0.776	0.378	1.77 (0.50–6.30)
6–10 years	1.064	0.479	4.942	0.026	2.91 (1.13–7.41)
>10 years	1.189	0.473	6.324	0.012	3.28 (1.30–8.29)
Hospitalization experience					
Never hospitalized*					
1–3 times	0.366	0.808	0.205	0.65	1.44 (0.30–7.02)
4–5 times	0.102	0.796	0.017	0.898	1.11 (0.23–5.28)
≥6 times	-0.304	0.879	0.119	0.730	0.74(0.13-4.14)
Experience with SGA-LAl					
No Experienced*					
Experienced	2.413	0.371	42.273	<0.001	11.11 (5.41-23.26)
Outpatient adherence					
Regular*					
Irregular	0.124	0.354	0.122	0.727	1.13 (0.57–2.26)
Age					
18~30*					
31~50	-0.893	1.037	0.743	0.389	0.41 (0.05–3.12)
51~65	-0.505	1.006	0.253	0.615	0.60 (0.08–4.33)
>65	-0.562	1.034	0.296	0.587	0.57 (0.08–4.32)

*Compared with this classification.

### 3.4 Patient cognition and concerns

Among the 155 patients willing to use SGA-LAIs, the most frequently endorsed perceived benefits were: avoiding daily medication (80.6%), enhancing efficacy (68.4%), improving quality of life (56.1%), and reducing the risk of relapse (54.8%).The most significant medication-related concerns in this cohort were:pain of injection pain (56.8%), concern about dependence (48.4%), the high price of medication (35.5%), and potential adverse drug reactions (30.3%). Conversely, among the 72 patients who were unwilling to consider SGA-LAIs, the predominant reasons for refusal, in descending order of frequency, included: rejection of needles (93.1%), rejection of changing change therapeutic regimens (79.2%), doubts about efficacy and adverse reactions (61.1%), and concerns about high expenses (25.0%).

## Discussion

In this study, we systematically evaluated the awareness, willingness to use, and associated influencing factors regarding SGA-LAIs among patients with schizophrenia who were receiving follow-up care at a psychiatric hospital. Our findings indicate that the overall willingness to use SGA-LAIs among the interviewed patients was 68.3%. Experience with SGA-LAIs and course of illness were identified as independent factors influencing willingness to use these formulations. Acceptance rates were significantly higher among patients who had prior medication experience and those with a longer disease course. Concurrently, this study revealed the principal barriers to patient acceptance, including limited recognition of the core advantages of SGA-LAIs, concerns related to medication use, and outright refusal of injectable treatments. These insights provide a targeted, empirical foundation for clinically optimizing strategies to promote SGA-LAIs.

Our results demonstrate a high level of acceptance of SGA-LAIs among outpatients with schizophrenia, which aligns with the findings of several studies. A survey by Mace et al. revealed that most patients with schizophrenia acknowledged the value of LAIs in ensuring accurate dosing and reducing rehospitalization rates (37). Research from Japan has indicated that patients currently receiving LAIs hold positive attitudes regarding side effects, relapse prevention, efficacy, injection-related pain, and costs, with acceptance significantly correlated with expectations for preventing relapse (38). Similarly, Blackwood et al. reported that 77% of patients with schizophrenia expressed a preference for LAIs over oral tablets (18). However, these findings contrast markedly with those of some studies conducted in China. A community-based survey in Shanghai revealed that only 3.16% of patients with schizophrenia desired to use LAIs (39), and another study in the Beijing community reported that only 18.1% of patients were willing to accept LAIs (33). The core reason for this discrepancy likely stems from sample heterogeneity: the respondents in our study were all outpatients under follow-up care at a tertiary psychiatric hospital.Compared with patients managed primarily in community settings, this population typically has a greater degree of illness insight, greater knowledge of treatment options, and more frequent exposure to professional medical information concerning SGA-LAIs (40). Variations in healthcare policies, alongside differences in the historical accessibility and market introduction timing of SGA-LAIs across regions, have historically constrained both patient and physician choices (41). Furthermore, 55.5% of the respondents in our study had prior experience using SGA-LAIs, a proportion considerably higher than that observed in community populations, and medication experience itself is a core factor enhancing patient acceptance. Additionally, in recent years, the continuous improvement in medical insurance coverage for SGA-LAIs in China and increased clinical promotion efforts may have collectively contributed to improved patient awareness and acceptance of these agents (42).

One of the core findings of this study is that prior experience with SGA-LAIs emerged as the strongest independent predictor of patient willingness to use this treatment modality. Compared with patients without such experience, patients with previous SGA-LAIs exposure demonstrated significantly greater willingness. These results underscore the pivotal influence of information exposure and direct, hands-on experience in fostering patient acceptance of novel therapeutic options. Furthermore, facilitating initial patient exposure to SGA-LAIs could serve as an effective strategy to increase the willingness to adopt this form of treatment (43). This observation aligns with the findings of several prior investigations. Systematic reviews have indicated that the most favorable attitudes toward LAIs are found among individuals who are currently using or have previously used these agents (44). Specifically, a willingness to use LAIs has been reported in 45% of patients with past LAI use and 75% of those currently on LAI therapy (45). A semistructured interview study involving community-dwelling patients with schizophrenia revealed that compared with those on oral medications, those treated with LAIs better recognize their specific advantages, such as eliminating the need to swallow pills daily or carry medication when leaving home (37). Similarly, an interview-based study employing grounded theory reported that despite a range of attitudes toward LAIs, the most positive perspectives consistently came from patients actively receiving LAI treatments (46). The underlying mechanism for this experience-dependent acceptance can be explained from two complementary perspectives. First, the most frequently endorsed advantage of SGA-LAIs among willing patients in this study was the “avoiding daily medication”. This benefit is fully appreciable only through direct treatment experience. Patients who have used LAIs experience a simplified treatment regimen and freedom from daily dosing concerns, which reinforces treatment acceptance. Previous work by Sugawara N et al. revealed that patients’ preferences for LAIs often stems from positive perceptions such as “not having to think about their medication” and “feeling no different from others” (38). Second, apprehensions among those without experience with LAIs often stem from subjective imagination and informational biases. For instance, while 93.1% of patients in the unwillingness group cited “rejection of needles” as a primary reason for refusal, 56.8% of patients in the willingness-positive group also expressed concern about injection pain; however, this concern did not deter their willingness. This contrast suggests that actual medication experience can substantially mitigate the irrational fears associated with injectable drug administration. Furthermore, misconceptions about pharmacotherapy, such as unfounded worries about “drug dependence” can be effectively addressed and corrected through structured doctor-patient communication (47). However, there may be even more unavoidable contradictions. For example, patients who are more familiar with their current oral treatment regimen may be either more willing to try an alternative (because they are actively engaged in their treatment) or more resistant to change (because they have become accustomed to their current therapy). Collectively, these findings indicate that enhancing patients’ initial exposure to and experience with SGA-LAIs could be a critical leverage point for improving prescription rates. It also reflects that, in real-world clinical practice, the experiences and information most patients receive regarding SGA-LAIs are predominantly positive. This highlights the significant role of effective clinical communication and evidence-based patient education in shaping treatment perceptions and decisions (48). Moreover, shared decision-making would improves patient’ acceptance. A cluster randomized trial showed that (13), compared with the control group receiving usual care (without emphasis on the use of LAIs), patients in the intervention group who were trained to encourage early use of LAIs had significantly shorter hospital stays. When an informed shared decision-making model was used to introduce LAIs, 86% of young early-stage patients expressed willingness to try the treatment, and 91% of them eventually accepted it.

Another significant finding from this study was that disease course was strongly positively correlated with patients’ willingness to use SGA-LAIs. Specifically, patients with a disease course exceeding five years demonstrated markedly greater acceptance of SGA-LAIs. These outcomes underscore the real-world need for sustained therapeutic strategies among individuals with prolonged schizophrenia. As the illness progresses, patients frequently experience repeated relapses and the cumulative burden of long-term oral medication regimens (49). Over time, they gain a more profound awareness of the chronic, persistent nature of the disorder and recognize the critical role of consistent treatment. As a result, these patients exhibit greater openness to LAI formulations, which can streamline treatment schedules and contribute to illness stability.This observation contrasts with findings from studies conducted in community-managed settings in China. For instance, Sun et al. (39) reported that individuals with shorter illness courses were more likely to use LAIs, a discrepancy that may be attributed to differences in sample sources. In community management settings, patients with shorter disease courses often enter such programs following acute-phase treatment, where healthcare providers may more actively recommend LAIs to prevent relapse. In contrast, among the outpatient population examined in this study, those with shorter illness course may have limited insight into the long-term management requirements of schizophrenia and may overestimate their adherence to oral medications (50), thereby reducing their motivation to switch to LAIs.These findings offer valuable guidance for targeted clinical communication. Patients with a disease course of more than five years represent a priority group for SGA-LAIs promotion. Proactive medication education should be directed toward this population, emphasizing core benefits such as simplified treatment regimens and reduced relapse risk.

In addition, this study delineates the core determinants underlying patient acceptance or reluctance toward SGA-LAIs, thereby establishing clear targets for clinical intervention. Willing patients prioritized “avoidance of daily oral dosing” and “enhanced therapeutic efficacy” which align with the principal clinical benefits of SGA-LAIs (51). Clinical education strategies should, therefore, emphasize these two advantages to address patients’ fundamental needs effectively.The primary concerns and barriers can be categorized into two groups. For patients willing to consider SGA-LAIs, the foremost concerns involved injection-related discomfort and fears of drug dependency. These concerns can be mitigated through refined injection techniques and by clarifying the critical distinction between “long-term medication necessary for disease management” and “substance dependence” (52). For patients who are currently unwilling to use LAIs, the dominant obstacles included a general aversion to injectable medications and resistance to altering their existing treatment regimen. These concerns underscores the importance of introducing information regarding SGA-LAIs earlier in the clinical management pathway. Specifically, interventions should aim to rectify misconceptions about injectable formulations, incorporate patients’ previous treatment experiences, and offer individualized assessments of the potential benefits of switching to LAIs. Adopting such a proactive, educational approach is advantageous over reserving LAIs solely for patients with documented adherence challenges (53).

The findings of this study have important implications for both clinical practice and health policy. At the clinical level, healthcare providers can more precisely identify priority populations for communication regarding SGA-LAIs, such as patients with a longer illness course, and enhance treatment acceptance through several targeted strategies. These include strengthening medication education, optimizing patients’ initial experience with LAIs, and directly addressing specific patient concerns (54). From a public health perspective, the results highlight actionable strategies to promote the broader clinical adoption of SGA-LAIs. Recommendations include improving medication accessibility, extending mental health education to outpatient and community settings, and reinforcing an integrated “hospital-community-family” management model (55). Implementing such measures could contribute to better long-term outcomes for individuals with schizophrenia and alleviate the social burden of the disease. Furthermore, it is essential to develop diversified and sustainable treatment support mechanisms. This involves expanding funding channels and enhancing the scope and intensity of supportive policies to provide more stable, long-term treatment assistance, thereby reducing treatment discontinuation due to financial barriers (56).

Although the present study focused on patient-level factors, emerging evidence suggests that clinicians’ attitudes, misconceptions about LAIs, and lack of familiarity with LAI dosing protocols also substantially influence actual prescription rates. For example, Weiden PJ et al. pointed out that clinicians may not introduce LAIs to patients in an optimal manner: either they assume that patients cannot overcome needle-related discomfort, or they present the treatment option with ambivalence rather than emphasizing its core advantages (57). Alternatively, clinicians often assume that patients will oppose the use of LAIs (58).

In this study, the odds ratio associated with previous SGA-LAIs experience is notably large. Several explanations may account for this strong association. First, direct experience or adequate knowledge of SGA-LAIs can substantially reduce fear and uncertainty toward an unfamiliar treatment, thereby greatly increasing acceptance. Second, residual confounding may have inflated the estimate. Unmeasured variables such as illness severity, insight, prior treatment satisfaction, and physician recommendation could be associated with both having prior SGA-LAIs experience and being willing to use SGA-LAIs. Third, selection bias may also play a role. As clinically stable outpatients may be more engaged in treatment, which could exaggerate the observed association. Furthermore, reverse causation cannot be ruled out. Patients who are already inclined to accept SGA-LAIs might proactively seek information or experience, rather than prior experience directly causing willingness.

Several limitations of this study should be noted, warranting cautious interpretation of the results. First, owing to its cross-sectional design, this study examined only the associations between the variables and could not establish causality. For instance, the observed relationship between illness course and medication willingness requires confirmation through prospective research. And residual confounding or selection effects may have contributed to the observed estimate. Second, the use of a single-center convenience sampling approach limits the generalizability of the findings. Participants were restricted to clinically stable outpatients from a tertiary psychiatric hospital, excluding inpatients, community-based patients lost to follow-up, and those in the acute phase, or populations with different health insurance coverage or socioeconomic backgrounds. Third, key variables such as illness severity, insight into illness, cognitive function, family support, and sources of health information were not assessed, which may introduce unmeasured confounding bias given their potential influence on medication use willingness. Additionally, a prior study indicated that psychiatrist’ attitudes toward LAIs can substantially affect patients’ acceptance (59), a factor not examined in this study. Fourth, this study did not analyze reasons for discontinuation among patients who had previously used but stopped SGA-LAIs, limiting a comprehensive understanding of how medication experience shapes willingness. Furthermore, grouping “uncertain” and”unwilling”responses into a single category may obscure meaningful differences in patient attitudes and thus affect the interpretation of the conclusions. Fifth, the questionnaire items were reviewed and adjusted by the research team together with an expert panel, who considered the items relevant. However, the limited size of the expert panel may have reduced the comprehensiveness of content validation.

Future studies should adopt multicenter, large-sample designs that include broader patient populations (e.g., community-dwelled samples) and employ more rigorous stratified sampling strategies to enhance the generalizability of the findings. In addition, qualitative methods could be integrated to explore the underlying mechanisms related to medication policies and clinical implementation of SGA-LAIs. To gain a more comprehensive understanding of patients’ willingness, future questionnaire items should be refined to capture key factors such as illness severity, cognitive level, prior experience with LAIs, family support, and clinician recommendations, as well as medication literacy and previous education on LAIs. Furthermore, willingness should be examined across different levels of prior knowledge and experience with SGA-LAIs, which would help reveal in more detail how familiarity with LAIs shapes treatment acceptance.

## Conclusion

This study demonstrated that patients with schizophrenia receiving follow-up care in psychiatric hospital outpatient clinics generally have a positive attitude toward the use of SGA-LAIs. Prior experience with SGA-LAIs and the course of illness emerged as independent determinants of patients’ willingness. Specifically, individuals with previous exposure to SGA-LAIs and those with a disease course exceeding five years showed significantly higher acceptance rates. Patients primarily perceive the core benefits of SGA-LAIs as reducing the burden of daily oral medication use and promoting clinical stability. In contrast, key barriers to adoption include apprehension toward injectable medications and reluctance to modify existing treatment plans. In clinical practice, it is advisable to prioritize psycho education for patients with longer illness courses. Targeted interventions should aim to increase the initial experiences with SGA-LAIs and address specific misconceptions. Implementing these strategies may improve long-term treatment outcomes and may facilitate the broader acceptance and implementation of SGA-LAIs in clinical practice.

## Data Availability

The original contributions presented in the study are included in the article/supplementary material. Further inquiries can be directed to the corresponding author.
